# Fish hosts, glochidia features and life cycle of the endemic freshwater pearl mussel *Margaritifera dahurica* from the Amur Basin

**DOI:** 10.1038/s41598-019-44752-9

**Published:** 2019-06-05

**Authors:** Ilya V. Vikhrev, Alexander A. Makhrov, Valentina S. Artamonova, Alexey V. Ermolenko, Mikhail Y. Gofarov, Mikhail B. Kabakov, Alexander V. Kondakov, Dmitry G. Chukhchin, Artem A. Lyubas, Ivan N. Bolotov

**Affiliations:** 1 Northern Arctic Federal University, Arkhangelsk, 163002 Russia; 2 Federal Center for Integrated Arctic Research, Russian Academy of Sciences, Arkhangelsk, 163000 Russia; 3 Saint-Petersburg State University, Saint-Petersburg, 199034 Russia; 4 A.N. Severtsov Institute of Ecology and Evolution, Russian Academy of Sciences, Moscow, 119071 Russia; 5 Federal Scientific Center of the East Asia Terrestrial Biodiversity, Far Eastern Branch of the Russian Academy of Sciences, Vladivostok, 690022 Russia

**Keywords:** Coevolution, Freshwater ecology

## Abstract

Margaritiferidae is a small freshwater bivalve family with 16 species. In spite of a small number of taxa and long-term history of research, several gaps in our knowledge on the freshwater pearl mussels still exist. Here we present the discovery of host fishes for *Margaritifera dahurica*, i.e. Lower Amur grayling, sharp-snouted lenok, and blunt-snouted lenok. The host fishes were studied in rivers of the Ussuri Basin. The identification of glochidia and fish hosts was confirmed by DNA analysis. The life cycle of *M*. *dahurica* and its glochidia are described for the first time. The SEM study of glochidia revealed that the rounded, unhooked *Margaritifera dahurica* larvae are similar to those of the other Margaritiferidae. *Margaritifera dahurica* is a tachytictic breeder, the larvae of which attach to fish gills during the Late August – September and finish the metamorphosis in June. Ancestral host reconstruction and a review of the salmonid - pearl mussel coevolution suggest that the ancestral host of the Margaritiferidae was a non-salmonid fish, while that of the genus *Margaritifera* most likely was an early salmonid species or their stem lineage. The overfishing of lenoks and graylings appears to be the most significant threat for this rare mussel species.

## Introduction

The study of parasites and hosts which have been connected to each other for tens of million years contributes significantly to our understanding of the evolutionary process^1–4^. A remarkable example of such associated species is presented in freshwater environments, in which freshwater mussels of the order Unionida (Bivalves) and fishes are closely connected to each other. Unionida, or naiades, use fishes as hosts for their larva (glochidium) during the parasitic stage. Our knowledge on the mussel-fish interactions is far from being complete, because host fishes of many mussel species are still unknown^5^. Such a knowledge gap leads to underestimating the role of fish in mussel conservation^6^ and hampers the development of effective mussel conservation strategies.

The freshwater pearl mussels (Margaritiferidae) is a small freshwater bivalve family with only 16 species^7^ but for less than half of these species the fish hosts are known. For instance, data on the hosts of *Margaritifera dahurica* (Middendorff, 1850), *Pseudunio homsensis* (Lea, 1865), *P*. *marocanus* (Pallary, 1918), and all five representatives of the genus *Gibbosula* Simpson, 1900 is absent or insufficient^7–11^. The identification of hosts is highly important for conservation of freshwater pearl mussels which are keystone species and ecosystem engineers in freshwaters. As other naiads, freshwater pearl mussels directly impact benthic processes as they burrow through sediments and ensure deposition of nutrients as filter feeders^12,13^.

One of the causes of freshwater pearl mussel population decline is the decreasing of abundance or local extinction of host fishes^14–19^. *M*. *dahurica* is listed in “The IUCN Red List of Threatened Species” under the “Data Deficient” category^20^. It means that reliable data on the population trends and abundance of this species is yet to be obtained. Our limited knowledge on the ecology and biology of *M*. *dahurica* enables to estimate possible threats correctly. For example, a relatively low number of juvenile mussels was recorded in several populations of *M*. *dahurica*^21^, but reasons for this phenomenon are yet to be studied.

**Figure 1:**
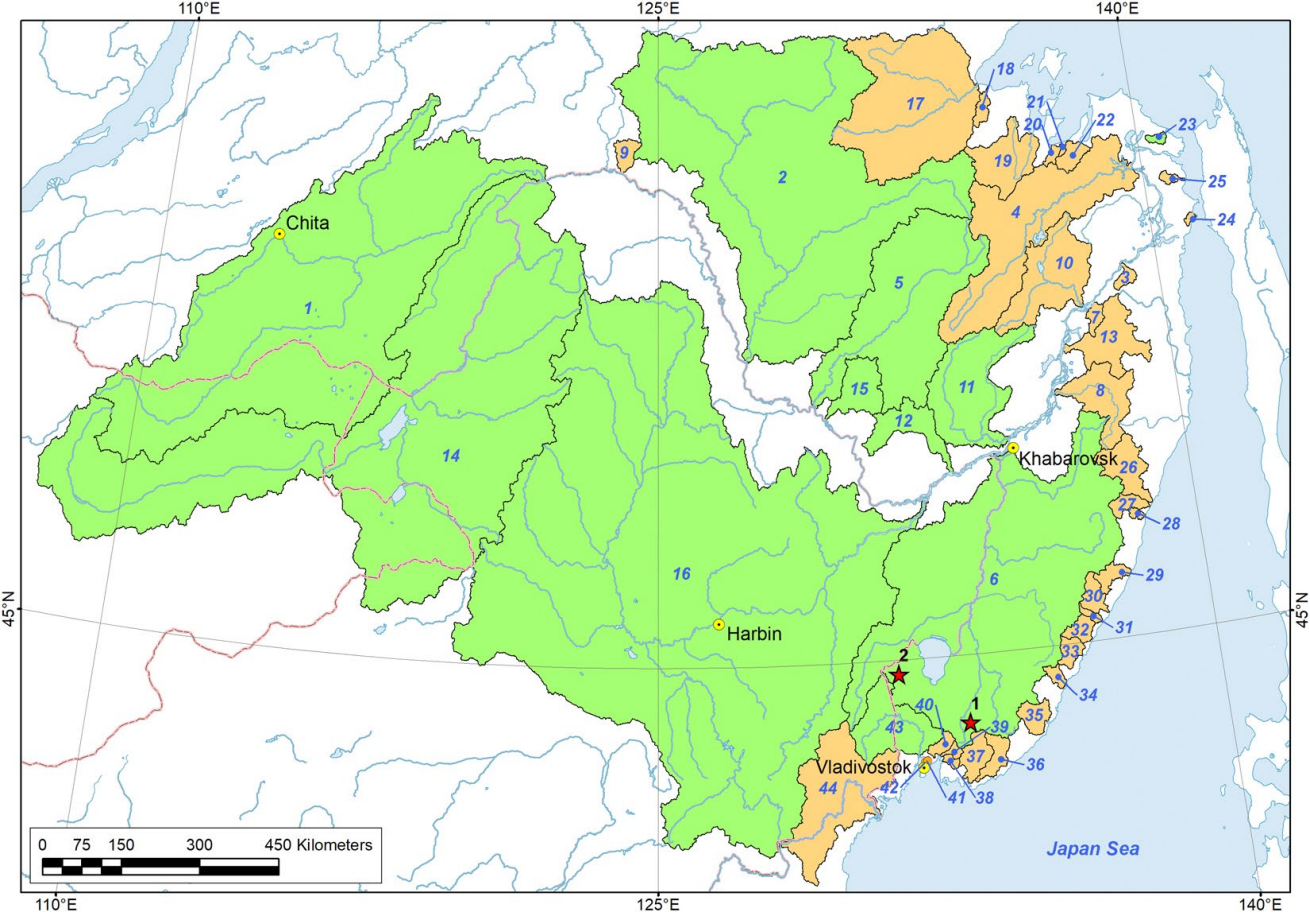
Distribution map of *Margaritifera dahurica* and its host fish genus *Brachymystax* in the Russian Far East. Blue numbers correspond to certain river basins (Supplementary Table 3). Green filling indicates co-occurrence of both the mussel and the host, and yellow filling indicates freshwater basins, in which only *Brachymystax* populations were recorded. Red asterisks indicate sites from which fish host samples were collected: (1) Muraveika River (tributary of the Ussuri River), and (2) Komissarovka River (tributary of the Khanka Lake). The map has been created using ESRI ArcGIS 10 software (www.esri.com/arcgis). The base of the map has been compiled from free open sources such as Natural Earth Free Vector and Raster Map Data (http://www.naturalearthdata.com), Global Self-consistent Hierarchical High-resolution Geography, GSHHG (http://www.soest.hawaii.edu/pwessel/gshhg), HydroSHEDS (Lehner, B., Verdin, K., Jarvis, A. (2008): New global hydrography derived from spaceborne elevation data. Eos, Transactions, AGU, 89(10): 93–94), and GADM (Global Administrative Areas (2012). GADM database of Global Administrative Areas, version 2.0. www.gadm.org).

*M*. *dahurica* ranges throughout the Amur River basin in the Russian Far East and China, and several adjacent separate rivers of the Okhotsk and Japan Sea drainages (Fig. 1). A single old record notes the occurrence of “*M*. *margaritifera*” in the north of the Korean Peninsula, but it is probably a misidentification of *M*. *dahurica*^22^, because distribution of *M*. *margaritifera* (Linnaeus, 1758) in Eurasia is restricted to Europe. The fish hosts of *M*. *dahurica* are yet to be discovered. Klishko and Bogan^23^ listed seven salmonid species in the Amur River drainage which could be the potential hosts for glochidia of this freshwater pearl mussel species. Masu Salmon *Oncorhynchus masou* (Brevoort, 1856) was suggested as a potential host in the Komarovka River (Razdolnaya Basin)^24^. Lenok or Manchurian trout *Brachymystax lenok* (Pallas, 1773) was observed to be infested by glochidia in the Komissarovka River (tributary of the Khanka Lake, Amur Basin) but the larvae were not identified with certainty^9^.The unknown reproductive timing of *M*. *dahurica* make the task of host identification complicated. Mussels use two basic reproductive strategies: bradytictic (long-term breeders) and tachytictic (short-term breeders) (Ortmann, 1919). Mussels with a bradytictic strategy spawn eggs to marsupia in the late summer, develop larvae during the fall and overwinter glochidia in the marsupia. Tachytictic breeders fertilize eggs during summer, and fully developed glochidia release from a host in the late summer or fall of the same year. Most of freshwater pearl mussels are tachytictic breeders, e.g. *Margaritifera margaritifera*^24^, *M*. *laevis* (Haas, 1910)^25^, *M*. *middendorffi* (Rosén, 1926)^26^, *M*. *falcata* (Gould, 1850)^27,28^, *M*. *hembeli* (Conrad, 1838)^29^ and *Pseudunio auricularius* (Spengler, 1793)^30^. *Cumberlandia monodonta* (Say, 1829) is the only example of a biannual spawner within the family^31^. However, for each breeding strategy the timing of life cycle stages is temperature dependent and can vary even within a species. For example, Bauer reported a *M*. *margaritifera* population in Germany, in which juvenile mussels leave a host in autumn, while in another population glochidia overwinter on a host^32^. The life cycle of *M*. *dahurica* is almost completely unstudied. However, it was reported that *M*. *dahurica* from the Ingoda River, Transbaikalia, Russia, starts to release glochidia when the water temperature become lower than 8–10 °C, which takes place at the end of September in the area^33^.

*M*. *dahurica* is the only species within the genus, the fish hosts of which are still unknown. Its nearest neighbor, *M*. *margaritifera*, use Salmonidae as a host for glochidia, like most of *Margaritifera* species^7^. It is logical to suppose that the larvae of *M*. *dahurica* also use salmonids as hosts. The lack of those data is the last white spot in our knowledge on fish hosts of freshwater pearl mussels parasitizing on salmonids. In the absence of these data general patterns of coevolution of freshwater pearl mussels and salmonids are debatable^24^ and cannot be resolved.

The aim of the present study is to describe the life cycle of *M*. *dahurica* and to improve our understanding of coevolution of the freshwater pearl mussels and their hosts. For this purpose, we: (1) identify the fish hosts of *M*. *dahurica*; (2) describe the morphology of the glochidium stage; (3) estimate reproductive timing of *M*. *dahurica*; and (4) reconstruct ancestral fish hosts for the freshwater pearl mussels on the basis of statistical approaches and multi-locus phylogeny.

## Results

### Discovery of fish hosts

**Figure 2:**
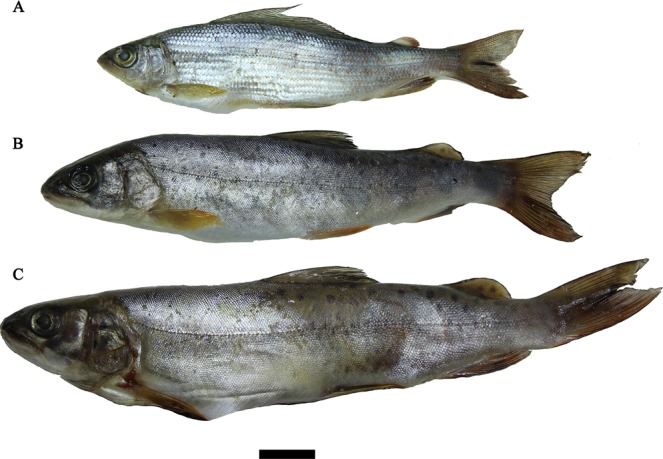
Hosts of *Margaritifera dahurica*, Komissarovka River, Khanka Lake Basin, March 2017, local fisherman leg. (**A**) Lower Amur grayling *Thymallus tugarinae*. (**B**) Sharp-snouted lenok *Brachymystax lenok*. (**C**) Blunt-snouted lenok *B*. *tumensis*. Scale bar = 20 mm. (Photos: Ilya V. Vikhrev).

Ten specimens of Lower Amur grayling *Thymallus tugarinae* Knizhin, Antonov, Safronov & Weiss, 2007 and one specimen of blunt-snouted lenok *Brachymystax tumensis* Mori, 1930 were caught in May 2017 in the Muraveika River, a tributary of the Ussuri River, Russian Far East (Fig. 1). Another two fish samples were collected in a small tributary of the Komissarovka River (tributary of the Khanka Lake) (Figs 1, 2). The first sample was caught in March 2017 and it contained eighteen sharp-snouted lenoks *Brachymystax lenok*, seven blunt-snouted lenoks, and one Lower Amur grayling. The second sample was caught in May 2017 and it contained eight blunt-snouted and three sharp-snouted lenoks. Two Lower Amur graylings were caught in another small unnamed tributary of the Komissarovka River. Both tributaries are close to each other.

**Figure 3:**
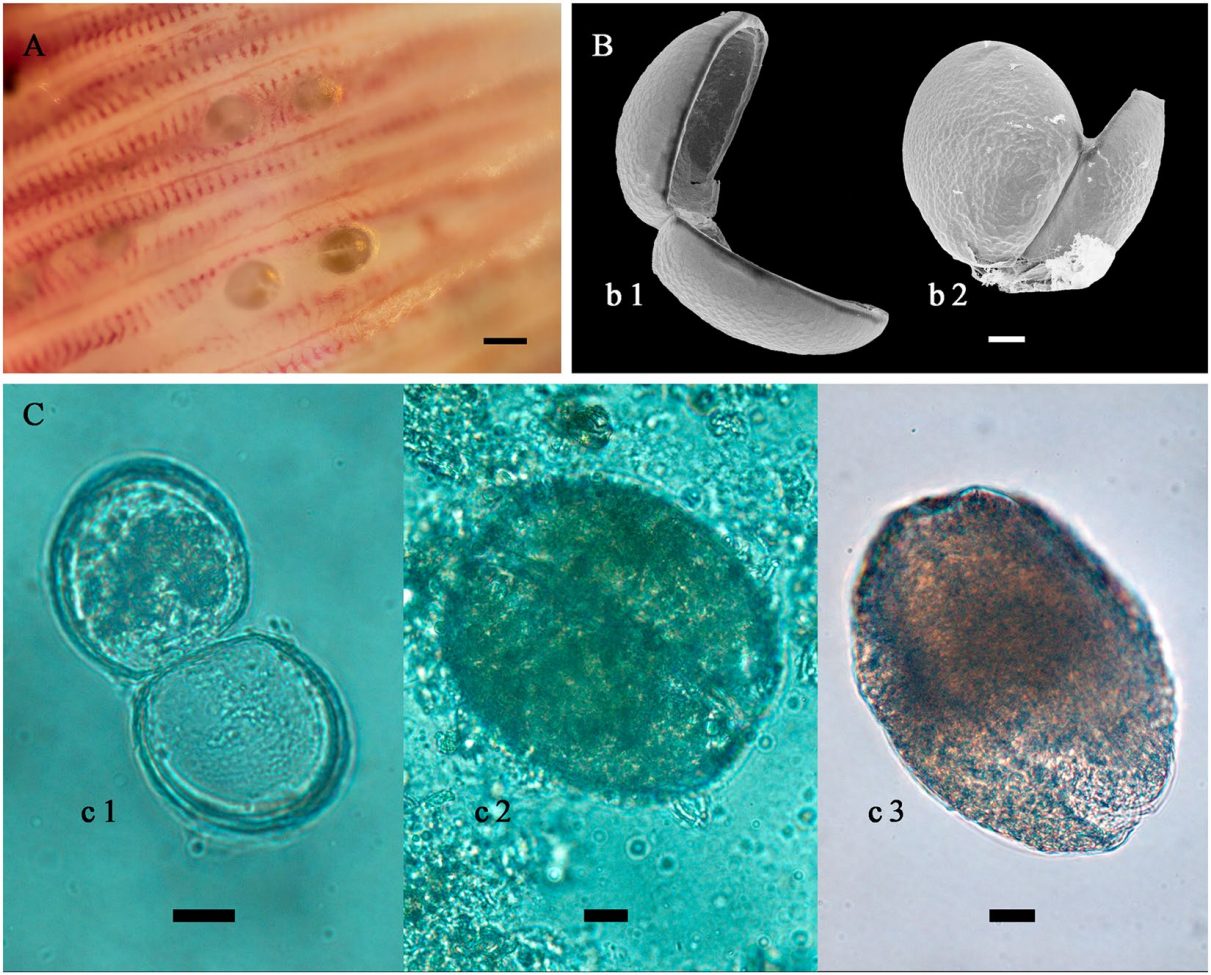
Morphology and growth of *Margaritifera dahurica* glochidia. (**A**) Glochidia on gills of *Brachymystax lenok*, Komissarovka River, 24 May 2017 (scale bar = 100 µm). (**B**) SEM microphotographs of glochidia, Tyrma River, Amur Basin, Eastern Siberia, 31 August 2015, including (**b1**) lateral view, and (**b2**) dorsal view (scale bar = 10 µm). (**C**) Glochidia during metamorphosis, including (**c1**) at the end of August (right after releasing), (**c2**) at March, and (**c3**) at Late May (scale bars = 20 µm). (Photos: Ilya V. Vikhrev [A, C] and Dmitry G. Chukhchin [B]).

Gills of all sampled fishes were examined for glochidia. The glochidia were identified using light microscopy on one of the ten Lower Amur graylings from the Muraveyka River, and on one of the three Lower Amur graylings from a tributary of the Komissarovka River. Both of these identifications were checked and confirmed by the DNA analyses (Table 1). Every lenok, that was caught in March 2017, was infested by glochidia according to the light microscopy investigation but was not checked by the DNA analysis. Among eleven lenoks caught in May 2017 in a tributary of the Komissarovka River, glochidia were identified, using light microscopy, on six fishes, i.e. on four blunt-snouted lenoks and two sharp-snouted lenoks. All these identifications were checked by the DNA analysis and the presence of glochidia was confirmed for five fishes. Glochidia on the sixth fish were recorded by microscopy, but the DNA identification was failed, likely due to a low number of the larvae. Encysted glochidia were attached to gill filaments and clearly visible on a fresh material (Fig. 3A). The COI and cyt-b partial sequences of infested fishes were examined to confirm species identification.

**Table 1:** Summary of DNA barcoding and morphological investigation of *Margaritifera dahurica* hosts and glochidia from tributaries of the Ussuri river basin [6]*.

Host fish species	Voucher no.	River	DNA barcoding of fish hosts, NCBI GenBank acc. no. for COI/cyt-b sequences	*M*. *dahurica* larvae	
				Microscopic investigation	DNA barcoding, NCBI GenBank acc. no.
**March 2017**					
*Brachymystax lenok*	2	Komissarovka	n/a	Abundant	n/a
*B*. *lenok*	3	Komissarovka	n/a	Abundant	n/a
*B*. *lenok*	4	Komissarovka	n/a	Rare	n/a
*B*. *lenok*	6	Komissarovka	n/a	Rare	n/a
*B*. *lenok*	8	Komissarovka	n/a	Rare	n/a
*B*. *lenok*	9	Komissarovka	n/a	Abundant	n/a
*B*. *lenok*	11	Komissarovka	n/a	Abundant	n/a
*B*. *lenok*	12	Komissarovka	n/a	Rare	n/a
*B*. *lenok*	13	Komissarovka	n/a	Abundant	n/a
*B*. *lenok*	15	Komissarovka	n/a	Abundant	n/a
*B*. *lenok*	16	Komissarovka	n/a	Rare	n/a
*B*. *lenok*	17	Komissarovka	n/a	Abundant	n/a
*B*. *lenok*	19	Komissarovka	n/a	Rare	n/a
*B*. *lenok*	20	Komissarovka	n/a	Abundant	n/a
*B*. *lenok*	21	Komissarovka	n/a	Abundant	n/a
*B*. *lenok*	22	Komissarovka	n/a	Rare	n/a
*B*. *lenok*	23	Komissarovka	n/a	Rare	n/a
*B*. *lenok*	24	Komissarovka	n/a	Rare	n/a
*B*. *tumensis*	1	Komissarovka	n/a	Rare	n/a
*B*. *tumensis*	5	Komissarovka	n/a	Rare	n/a
*B*. *tumensis*	7	Komissarovka	n/a	Rare	n/a
*B*. *tumensis*	10	Komissarovka	n/a	Rare	n/a
*B*. *tumensis*	14	Komissarovka	n/a	Rare	n/a
*B*. *tumensis*	18	Komissarovka	n/a	Rare	n/a
*B*. *tumensis*	25	Komissarovka	n/a	Rare	n/a
*T*. *tugarinae*	1	Komissarovka	n/a	Absent	n/a
**May 2017**					
*Brachymystax lenok*	gil 18/9	Komissarovka	MG951555/MK024300	Abundant	MK024308
*B*. *lenok*	gil 19/7	Komissarovka	MG951555/MK024301	Abundant	MK024309
*B*. *lenok*	gil 22/3	Komissarovka	n/a	Absent	n/a
*B*. *tumensis*	gil 22/4	Komissarovka	MG951556/MK024305	Abundant	MK024313
*B*. *tumensis*	gil 22/8	Komissarovka	MG951556/MK024306	Rare	MK024314
*B*. *tumensis*	gil 22/10	Komissarovka	n/a	Absent	n/a
*B*. *tumensis*	gil 22/1	Komissarovka	n/a	Absent	n/a
*B*. *tumensis*	gil 22/11	Komissarovka	n/a	Rare	n/a
*B*. *tumensis*	gil 17/2	Komissarovka	MG951556/MK024299	Abundant	MK024307
*B*. *tumensis*	gil 22/5	Komissarovka	n/a	Absent	n/a
*B*. *tumensis*	gil 22/6	Komissarovka	n/a	Absent	n/a
*B*. *tumensis*	gil 20/2	Muraveika	MG951557/n/a	Absent	n/a
*Thymallus tugarinae*	gil 20/3	Muraveika	n/a/MK024302	Rare	MK024310
*T*. *tugarinae*	gil 20/4	Muraveika	n/a	Absent	n/a
*T*. *tugarinae*	gil 20/5	Muraveika	n/a	Absent	n/a
*T*. *tugarinae*	gil 20/6	Muraveika	n/a	Absent	n/a
*T*. *tugarinae*	gil 20/7	Muraveika	n/a	Absent	n/a
*T*. *tugarinae*	gil 20/8	Muraveika	n/a	Absent	n/a
*T*. *tugarinae*	gil 20/9	Muraveika	n/a	Absent	n/a
*T*. *tugarinae*	gil 20/10	Muraveika	n/a	Absent	n/a
*T*. *tugarinae*	gil 20/11	Muraveika	n/a	Absent	n/a
*T*. *tugarinae*	gil 20/12	Muraveika	n/a/MK024303	Absent	MK024311
*T*. *tugarinae*	gil 21/1	Komissarovka	n/a/MK024304	Rare	MK024312
*T*. *tugarinae*	gil 21/2	Komissarovka	MG951555/MK024300	Absent	n/a

*River basin numbers given in brackets correspond to those in Fig. 1 and Supplementary Table 3. n/a – Not available.

### Molecular identification of glochidia

DNA barcoding of glochidia observed on the fish gills reveals that Lower Amur grayling, sharp-snouted lenok and blunt-snouted lenok are host species for *M*. *dahurica* (Table 1). Among 11 lenoks caught in May in the Komissarovka river, infestation of 5 lenoks was confirmed with certainty thanks to the high concentration of glochidia DNA in the samples. Other fishes had a weak signal which did not allow us to say unambiguously whether it is the mussel DNA or not. Among 12 sampled Lower Amur graylings only three fishes were infested according to the DNA barcoding analysis (Table 1).

### Morphological identification of glochidia

Unhooked glochidia of *M*. *dahurica* have D-shaped form with straight hinge and rounded ventral margin (Fig. 3B). The length of released glochidia (mean 57.4 µm, SD ± 3.5 µm, *N* = 26) is comparable to those in the other Margaritiferidae (Supplementary Tables 1 and 2). The glochidial surface has small depressions covering over the shell without any structures or strong sculpture. The comparison of released glochidia and glochidia at the end of metamorphosis reveals that the mean length of larvae is increasing in more than two times during the metamorphosis (Fig. 3C; Supplementary Table 2).

### Life cycle of *Margaritifera dahurica*

**Figure 4:**
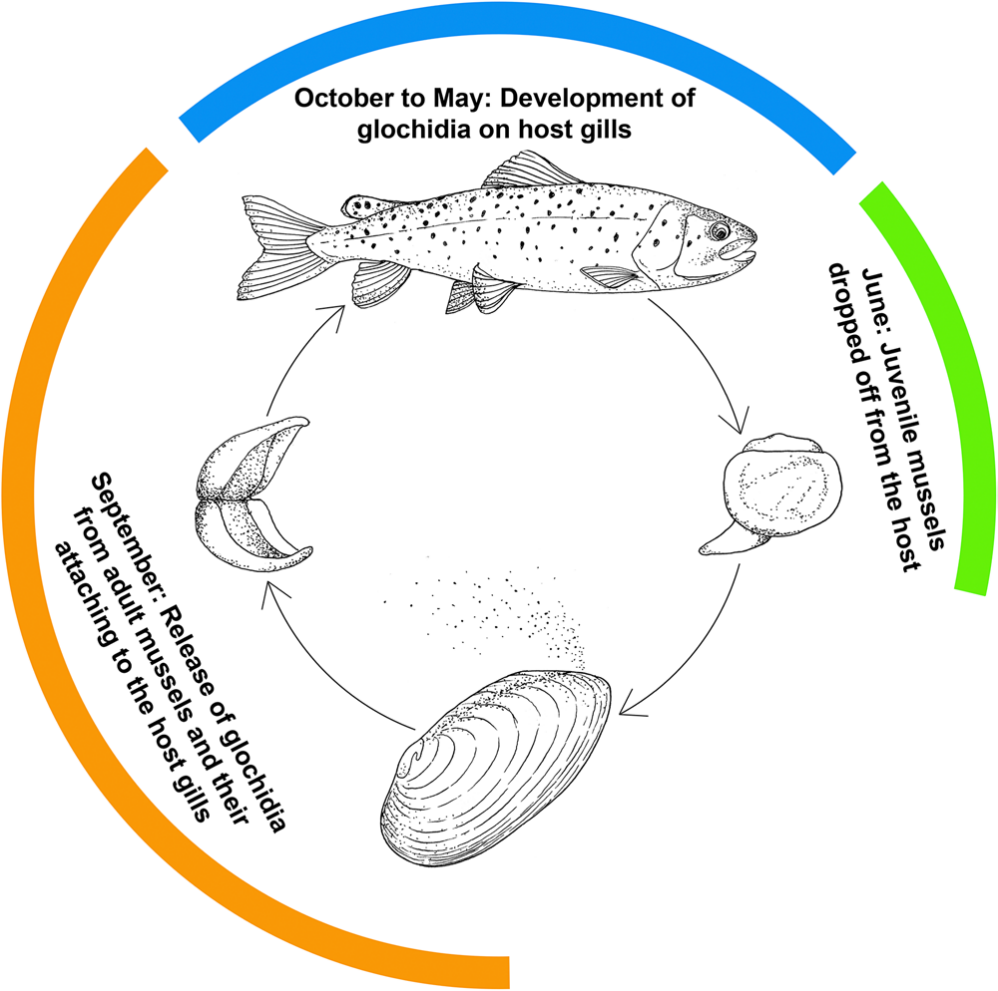
Simplified scheme of *Margaritifera dahurica* life cycle (See details of field observations in Table 2). The host fish are *Brachymystax lenok*, *B*. *tumensis* and *Thymallus tugarinae*. (Graphics: Inga S. Paltser).

General scheme of the life cycle of *M*. *dahurica* is very similar to that of *M*. *margaritifera* (Fig. 4; Table 2). The glochidia of the latter are discharged into a river in mid- to late summer and developed in the host gills for a period up to 10 months^34^. Mature *M*. *dahurica* collected in the mid-July had empty marsupia. Aborted glochidia were collected at the end of August from the individuals that were disturbed during measurements, which means that specimens were ready to release glochidia in the nearest future. Encysted glochidia were observed in both fish samples from March and May. Some of glochidia registered in March were of similar size as free-living larvae after releasing from mussels, and others were up to four times larger. Glochidia observed at the end of May have had less variable length from 89.4 µm to 189.1 µm (min-max values). We found significant size differences between the samples of glochidia collected in September, March and May (Kruskal-Wallis test: H = 57.4, p < 0.0005) with the mean length of 57.4 µm, 92.2 µm and 150.7 µm, correspondingly.

**Table 2:** Field observations of life cycle of *Margaritifera dahurica* and comparison of glochidia release timing in the northern and southern parts of the range.

River*	Drainage	Date	Observations	Life cycle stage
Ussury River [6]	Amur River	20 July 2012	Empty marsupia	Eggs maturation in gonades
Tyrma River [5]	Amur River	31 August 2015	Mature glochidia in marsupia and glochidia releasing from disturbed mussels	Mussels are ready to release glochidia
Ingoda River [1]**	Ditto	End of September	Ditto	Ditto
A tributary of the Komissarovka River [6]	Khanka Lake = > Amur River	March 2017	Glochidia of different size on the host gills: difference between the smallest and the biggest larva is more than four times	Growth of glochidia
A tributary of the Komissarovka River [6]	Khanka Lake = > Amur River	25 May 2017	Glochidia on the host gills: still different in size, but difference between the smallest and the biggest larva is around two times	Glochidia near to the end of metamophosis

*River basin numbers given in brackets correspond to those in Fig. 1 and Supplementary Table 3.

**Published observation^33^.

### Distribution range of *Margaritifera dahurica* and its hosts

A review of the body of literature shows that the distribution range of *M*. *dahurica*^35^ fully agrees with those of lenoks (Fig. 1, Supplementary Table 3). The two lenok species have been recognized as two different species recently, and we therefore cannot compare the distribution of *M*. *dahurica* and every of the two lenok species separately. The lenoks were observed in all the regions in which *M*. *dahurica* was recorded (Fig. 1, Supplementary Table 3). These fishes inhabit the Amur River drainage within the Russian territory^36,37^, China^38^ and Mongolia^39^. The lenoks were also recorded in rivers of the southern part of the Okhotsk Sea coast and the Russian part of the Japan Sea coast^40,41^, e.g. in the Razdolnaya River^42^.

### Ancestral host reconstruction

**Figure 5:**
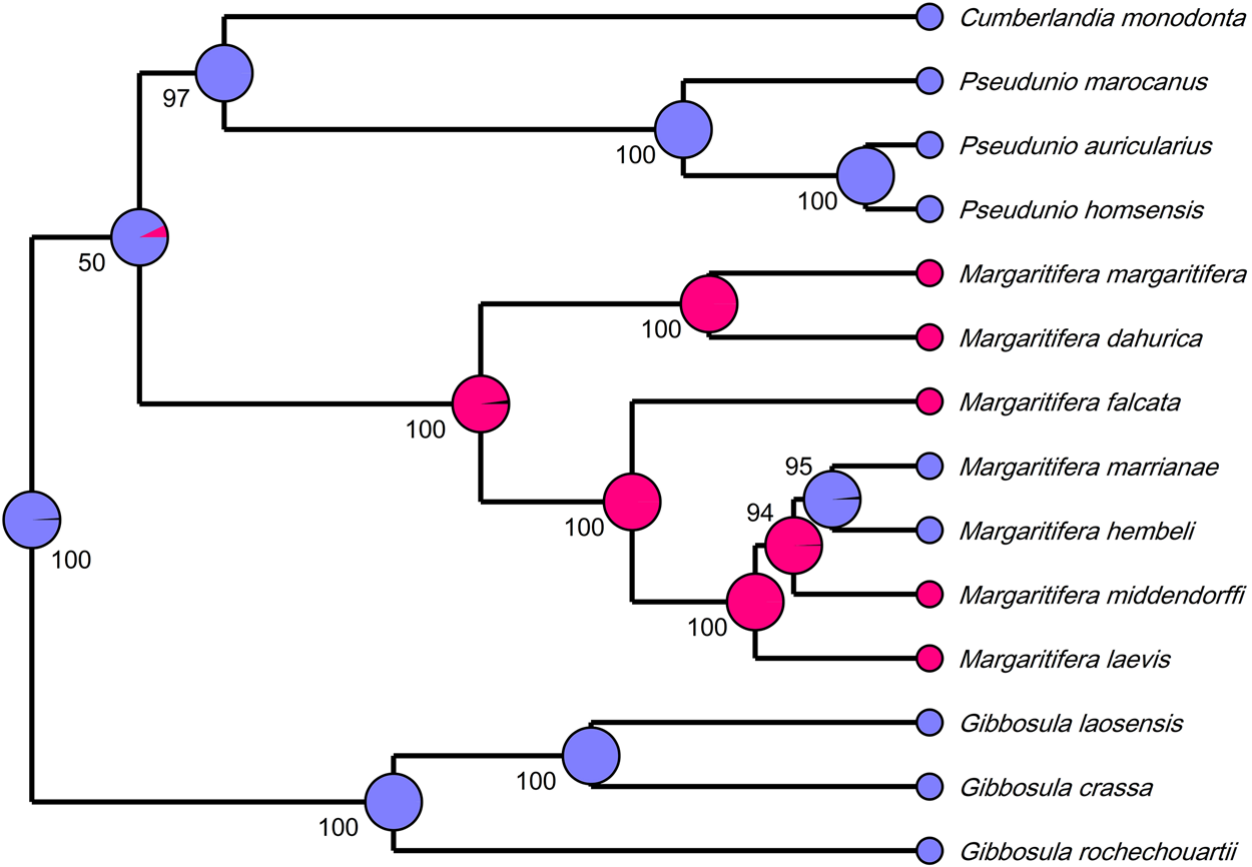
Ancestral host reconstruction for the Margaritiferidae on the basis of Bayesian MCMC analyses. The statistical models were calculated using a multi-locus fossil-calibrated phylogeny and fish host database of Lopes-Lima *et al*.^7^. Pink color indicates salmonid hosts, and violet color indicates non-salmonid hosts. Numbers near nodes are Bayesian posterior probabilities.

Our Bayesian Markov chain Monte Carlo (MCMC) modeling suggests that the ancestral host of the Margaritiferidae was a non-salmonid fish (probability = 98.9%) (Fig. 5). In contrast, the ancestral host of the genus *Margaritifera* most likely was an early salmonid species or their stem lineage (probability = 97.8%). The hypotheses of salmonid hosts of the most recent common ancestor (MRCA) of *M*. *margaritifera* + *M*. *dahurica* subclade and of the “Pacific” subclade were also fully supported by this model (probability = 100%) (Fig. 5). The interactions of *M*. *hembeli* and *M*. *marrianae* with non-salmonid hosts appear to be a secondary adaptation derived from a common ancestor of both species in North America (probability = 98.4%).

## Discussion

### Morphology of glochidia and life cycle of *Margaritifera dahurica*

General patterns of glochidia morphology of *M*. *dahurica* are similar to those in the other Margaritiferidae which have unhooked, rounded, small larvae against hooked, triangular and larger larvae in the Unionidae^43^. Despite the close genetic relationship to *M*. *margaritifera*, glochidia of *M*. *dahurica* are morphologically more similar to the larvae of *P*. *auricularius*, *M*. *laevis* and *M*. *middendorffi*, due to their rounded ventral margin^44,45^ against the slightly more triangular ventral margin of *M*. *margaritifera* larvae^46^. Regarding size, the glochidia of *M*. *dahurica* fit well within the size range of larvae of other freshwater pearl mussels, with exception of *P*. *auricularius* having the largest larvae among the Margaritiferidae (Supplementary Table 1).

Our findings reveal that *M*. *dahurica* is a tachytictic breeder with a long-term parasitic stage which passes throughout the winter (Fig. 4). According to Klishko^33^, *M*. *dahurica* starts to release glochidia when water temperature drops below 8–10 °C, which occurs at the end of September in Eastern Siberia. It is well known that reproductive timing in freshwater mussels shifts depending on water temperature^25,27,28^. In northern regions the glochidia release starts earlier, as we observed for *M*. *dahurica* in the Tyrma River, north of Khabarovsky Kray (Table 2). The fact that the life cycle timing of *M*. *dahurica* is very similar to that of *M*. *margaritifera* is in agreement with the phylogenetic proximity of two species^47^. Representatives of the genus *Thymallus* have not yet been considered as potential hosts for the larvae of freshwater pearl mussels. Our discovery of *M*. *dahurica* glochidia parasitizing on *Thymallus tugarinae* expands the current knowledge on the host range in the Margaritiferidae.

Our study describes only general patterns of the life cycle of *M*. *dahurica*. However, the details are still incomplete and are in need of future studies. The data such as the age of maturity, dependence between temperature and timing of juveniles drop off from the host, certain dates of life cycle stages for different regions, and testing other putative host fishes, e.g. Siberian Taimen *Hucho taimen* (Pallas, 1773), are of great importance for the effective conservation planning for *M*. *dahurica*.

### Co-occurrence of *Margaritifera dahurica* and the *Brachymystax* species

Our results reveal that the host-parasite interaction between *M*. *dahurica* and lenoks corresponds well to the published data on the distribution and habitat preferences of these fish taxa (Supplementary Table 3). Sandy-gravel and gravel-pebble grounds at riffles and runs, typical habitats of the freshwater pearl mussels^35^, are also inhabited by lenoks^48^. However, lenoks share a wider distribution than *M*. *dahurica*. Besides the regions inhabited by the freshwater pearl mussel, these fishes are distributed in Siberia, Korean Peninsula^49,50^, and in several Eastern Chinese rivers^51^. The sharp-snouted lenok and the blunt-snouted lenok differentiated well by molecular data and morphology, i.e. shape of the head, number of gill rakers and osteological characters. Both species coexist in sympatry through the range including the Amur Basin with the Komissarovka and Myraveika rivers. Taxonomic position and phylogeny of these two lenok forms are actively discussed^41,52–57^, however we consider blunt- and sharp-snouted forms as two separate species based on significant genetic differences even in sympatric populations. Lenok from rivers of the Qinling Mountains, belonging to the Yangtze and Yellow River basins in China, was initially described as the subspecies *Brachymystax lenok tsinlingensis* Li, 1966, but was recently elevated to the species level based on molecular data^58^. This taxon also inhabits south of the Korean Peninsula^50^.

### Coevolution of salmonid fishes and freshwater pearl mussels

Fossil records indicate that representatives of the family Margaritiferidae were abundant in Mesozoic water bodies of Southern Laurasia^7,47^ and in the north of modern Africa^59,60^. Meanwhile, it is considered that salmonids originated not earlier than in the Cenozoic era^61,62^, and Mesozoic freshwater pearl mussels, most likely, used other hosts, like the recent Margaritiferidae from southern regions (Table 3 and Fig. 5).

**Table 3:** Review of suitable hosts of the freshwater pearl mussels (Margaritiferidae).

Mussel species	Fish hosts
*Margaritifera dahurica*	Salmonidae: sharp-snouted lenok (*Brachymystax lenok*), blunt-snouted lenok (*B*. *tumensis*), and Lower Amur grayling (*Thymallus tugarinae*) [this study]
*Margaritifera margaritifera*	Salmonidae: Atlantic salmon (*Salmo salar*), brown trout (*S*. *trutta*), Arctic charr (*Salvelinus alpinus*), brook trout (*S*. *fontinalis*) (high susceptible host in North America but less susceptible or even an unsuitable host fish in Europe), and Danube salmon (*Hucho hucho*) (less susceptible host)^7,24,68,90,91^
*Margaritifera falcata*	Salmonidae: Chinook salmon (*Oncorhynchus tshawytscha*), Coho salmon (*O*. *kisutch*), cutthroat trout (*Parasalmo clarki*), and steelhead trout (*P*. *mykiss*); Gasterosteidae: three-spined stickleback (*Gasterosteus aculeatus*)^92^
*Margaritifera hembeli*	Esocidae^7^
*Margaritifera marrianae*	Esocidae^7^
*Margaritifera laevis*	Salmonidae: Masu salmon (*Oncorhynchus masou*)^25,45,93,94^
*Margaritifera middendorffi* (=*M*. *togakushiensis*)	Salmonidae: White-spotted char (*Salvelinus leucomaenis*)^95^
*Cumberlandia monodonta*	Hiodontidae: Mooneye (*Hiodon tergisus*) and Goldeye (*H*. *alosoides*)^96^
*Pseudunio auricularius*	Acipenseridae: sturgeons (*Acipenser* spp.); Blenniidae: River blenny (*Salaria fluviatilis*); Gasterosteidae: Three-spined stickleback (*Gasterosteus aculeatus*)^7,97–99^
*Pseudunio homsensis*	Unknown but surely non-salmonids (according to the distribution range)^100^
*Pseudunio marocanus*	Unknown, probably non-salmonids [M. Lopes-Lima, pers. comm. 2018]
*Gibbosula laosensis*	Unknown but surely non-salmonids (according to the distribution range)^14^
*Gibbosula crassa*	Unknown but surely non-salmonids (according to the distribution range)^7^
*Gibbosula rochechouartii*	Unknown but surely non-salmonids (according to the distribution range and habitats)^11^
*Gibbosula confragosa*	Unknown^7^
*Gibbosula polysticta*	Unknown^7^

The most ancient fossil representatives of the genus †*Eosalmo* (earliest known salmonids), that were found in British Columbia^62^ and Kamchatka^61^, document that ancient salmonids existed in the northern part of the Pacific Ocean drainage in the Eocene. This record corresponds well with the time of appearance of freshwater pearl mussels in the region. These mussel taxa appeared at the northern coast of the Pacific Ocean for a first time near the Paleocene – Eocene boundary^7^. †*Margaritifera herrei* (Hannibal, 1912) was spread in waterbodies located on the territory of the modern California^63^, while †*M*. *perdahurica* (Yokoyama, 1932), †*M*. *otatumei* (Suzuki, 1942) and †*M*. *owadaensis* Noda, 1970 inhabited territory of the modern Hokkaido Island^47^. Ziuganov *et al*.^24^ hypothesized that spreading of freshwater pearl mussels across the northern part of the Pacific drainages was likely related to the adaptation to parasitizing on salmonids.

During the mid-Eocene epoch (~42 Ma), freshwater pearl mussels appeared on the territory of the modern Yakutia, and then spread further to the west and south^7^. Moving southward, at the mid-Oligocene (~28 Ma), freshwater pearl mussels colonized the area of the modern Aral Sea and southeast shore of Lake Baikal. These freshwater pearl mussels were considered to be a single species, †*M*. *martinsoni* Modell, 1964^47^. Remarkably, *Brachymystax* representatives appeared in paleontological records in the Upper Oligocene, e.g. †*B*. *bikinensis* Sytchevskaya,1986 was discovered in the Ussuri River drainage^61^. Today, lenoks still inhabit the rivers of Yakutia^53^ and the Baikal Lake Area^39^. Therefore, it is likely that lenoks were hosts of glochidia of *M*. *martinsoni*. The fact that endemic trout (*Salmo trutta oxianus* Kessler, 1874) inhabits the Aral Sea drainage^64^, can be an indirect evidence that ancestors of the genus *Salmo* might be possible hosts for the larvae of *M*. *martinsoni* in this area. The data on the distribution of *M*. *martinsoni* and its putative host fish supports the hypothesis that this fossil taxon could be considered the MRCA of *M*. *dahurica* and *M*. *margaritifera*^47^.

As it has been shown recently, *M*. *dahurica* forms a subclade together with *M*. *margaritifera*, which is distinct from a subclade containing the other freshwater pearl mussel species from the Pacific Ocean drainage^7,47,59^.The origin of *M*. *margaritifera* cannot be tracked by available paleontological data. However, Chepalyga^65^ suggested that the fossil species †*M*. *arca* Chepalyga, 1965, described from the Upper Pliocene of the Dniester and Danube river valleys, can be an ancient stem lineage of *M*. *margaritifera*.

The primary hosts of *M*. *margaritifera* are Atlantic salmon *Salmo salar* Linnaeus 1758, brown trout *Salmo trutta* Linnaeus 1758, and Arctic charr *Salvelinus alpinus* (Linnaeus 1758) (Table 3). Like †*M*. *arca*, representatives of the genus *Salmo* also appeared in paleontological records in the Late Pliocene - Pleistocene. For example, †*Salmo derzhavini* Vladimirov, 1946 inhabited the territory of the modern Armenia^66^. Likely, ancestors of *M*. *margaritifera* migrated to Europe together with ancestors of *Salmo* or *Hucho*, distributed in Europe, Siberia, and the Far East of Russia. However, *Hucho* appeared in Europe much earlier than †*M*. *arca*, i.e. in the late Miocene^67^. Moreover, the Danube salmon *Hucho hucho* (Linnaeus, 1758) was recorded as a least suitable host of *M*. *margaritifera* glochidia^68^. At the same time, Siberian Taimen *H*. *taimen* can be considered a potential host for the larvae of *M*. *dahurica* as far as taimens are close to lenoks by morphological and molecular data^41^. Balakirev *et al*.^69^ even recorded a genetic introgression between these two genera. However, this hypothesis needs to be confirmed by means of an experimental approach.

Numerous studies are dedicated to investigation of molecular phylogeny of salmonids, in particular evolutionary interactions between the genera *Brachymystax*, *Hucho*, and *Salmo*^70^. Osinov and Lebedev^71^, using a large dataset of protein-coding genes, revealed that *Brachymystax*, *Hucho* and *Salmo* belong to the same clade. An analysis of amino acid partial sequences of the COI returned the same result^70^. A genetic proximity of *M*. *margaritifera* and *M*. *dahurica* may be evidence of close evolutionary relations of their host-fishes, and correspond well to the findings of Osinov and Lebedev^71^ and Artamonova *et al*.^70^.

### Implications for conservation of *Margaritifera dahurica* and salmonids in the genus *Brachymystax*

Most populations of *M*. *dahurica* inhabit clean mountain rivers in remote areas where sources of industrial, agricultural and recreational pollution are still lacking. Shell and pearl harvesting from *M*. *dahurica’s* was praticed in the IX–V centuries BC^72,73^, but was prohibited in Russia in 1964^74^. Nowadays only illegal harvesting occurs occasionally in small amounts (personal observations). River damming bears a potential risk for several populations, but this threat requires additional research. Parasitizing of bitterling (*Rhodeus sericeus* (Pallas, 1776), Cyprinidae) embryos in gills of *M*. *dahurica* was mentioned among biological threats for this mussel species^23^.

Now that, hosts of glochidia of *M*. *dahurica* have been discovered, it has become became clear that one of the main threats for this species is population decline of the *Brachymystax* species. *Brachymystax lenok* is listed as an endangered species in the “Red List of China’s Vertebrates”^75^, and as a vulnerable species in the “Mongolian Red List of Fishes”^76^ and “Korean Red List of Threatened Species”^77^. A few populations are listed in the “Red Data Book of the Russian Federation”^78^ under the “endangered group of populations” category. All these red lists unite blunt- and sharp-snouted lenoks under the name *Brachymystax lenok*. Declines in the abundance of lenoks due to overfishing, water pollution and environmental changes^79^, in particular, destruction of their natural habitats caused by channel improvement^77^, was recorded in China. The main threat for lenoks in rivers of Russia is illegal fishing, which is especially intensive in small rivers of the Japan Sea basin in the Primorye Region^48^.

Finally, artificial breeding of lenoks is well developed at the present time^80–82^. We suggest that the artificial breeding of *M*. *dahurica* at fish hatcheries together with lenoks can be a possible approach for the future conservation of this freshwater pearl mussel species.

## Methods

### Data sampling

Glochidia were collected prior to the start of metamorphosis, right after release from mussels that were disturbed during measurements in the Tyrma River (Amur Basin) near the Tyrma settlement (50.0159°N, 132.1271°E) on 31 August 2015 and fixed in 96% ethanol. Sampling of the prospective host fishes was performed in March and at the end of May 2017 in the Muraveika River (Ussuri Basin) near the Elovka village (43.8432°N, 133.2160°E) and in two tributaries of the Komissarovka River (Khanka Lake Basin) near the Barabash-Levada village (44.7641°N, 131.4217°E). Rivers are located in the Primorye Region, Russian Far East. Investigated fishes were caught by a local fisherman using fishing net. Fishes that were caught in March were kept frozen until gills examination in the laboratory. Gills of every fish from all samples were examined by light microscopy. After that, a first gill arc was fixed in 5% formaldehyde to allow counting of the number of gill rakers. The rest of the fish gills, as well as snips of fish muscle soft tissues, were fixed in 96% ethanol for further DNA analyses. Fixed material was deposited in the collection of the Russian Museum of Biodiversity Hotspots (RMBH), Federal Center for Integrated Arctic Research, Russian Academy of Sciences, Arkhangelsk, Russia.

### Species identification of fishes and glochidia by molecular analysis

Fish gills of Lower Amur grayling (*Thymallus tugarinae*), blunt-snouted lenok (*Brachymystax tumensis*) and sharp-snouted lenok (*Brachymystax lenok*) infested by glochidia were taken for species identification of the mussel larvae (Table 1). Samples were stored in 96% ethanol (1:5) and DNA was extracted using the phenol/chloroform extraction procedure^83^. The COI partial sequences of glochidia were amplified by polymerase chain reaction (PCR) using the universal primer LCO1490^84^ and our own designed primer MarMIDL-R (5′-GAAAAAATAGCCAAGTCTACAG- 3′). The primer MarMIDL-R was used to obtain a short PCR product (423 bp) that allows examination of partly degraded DNA. Fish identification was performed by amplification of the COI partial sequences using the universal primers FishF2 and FishR2^85^, and cyt-b partial sequences using the universal primers Glu L and Thr H^86^. Amplification was performed using 25 μl of a buffer solution (Fermentas) (75 mM Tris-HCl (pH 8.8), 20 mM (NH_4_)_2_SO_4_, 0.1% Tween 20, and 2 mM MgCl_2_). The PCR mix contained approximately 200 ng of total cellular DNA, 10 pmol of each of two (forward and reverse) primers, 200 μmol of each dNTP and 0.8 units of Taq DNA polymerase (SibEnzyme Ltd., Novosibirsk, Russia). Thermocycling included the initial denaturation stage (4 min at 95 °C) followed by 34–38 cycles consisting of denaturation at 95 °C (50 sec), annealing at 58 °C (50 sec), and extension at 72 °C (50 sec) followed by a final extension step at 72 °C (5 min). Forward and reverse sequencing was performed on an automatic sequencer (ABI PRISM 3730, Applied Biosystems) using the ABI PRISM BigDye Terminator v. 3.1 reagent kit in the Institutional Collective Usage Facility “Genome” of the Institute of Molecular Biology of the Russian Academy of Sciences. The resulting sequences were checked using a sequence editor (BioEdit v. 7.2.5^87^). The obtained sequences were compared with those from NCBI GenBank database using BLAST (www.ncbi.nlm.nih.gov).

### Morphological studies of glochidia

The morphology of free-living glochidia (*N* = 144) and encysted larvae (*N* = 35) on fish gills were studied using light and scanning electron microscopy. Fresh gills with encysted glochidia were photographed with a Canon EOS 7D camera (Canon Inc., Japan) with a reversed Jupiter 37 A 70 mm lens (KOMZ, Russia) mounted on the top of a microscope (LOMO C1Y4.2, Russia). Preparation of the fixed glochidia for microscopy followed the general scheme described by Hoggarth^88^ with our modifications. Glochidial mass stored in 96% ethanol was washed by deionized water and kept at 57 °C in a solution of 390 ml phosphate buffer (pH = 6.86) and 10 mk proteinase K (concentration = 4 mg/ml). The samples were inspected every 15 min and removed from the thermostat when the first glochidia with open valves were observed. Thereafter glochidia were washed with deionized water and stored in 96% ethanol prior to examination. Length, height and width measurements of free-living glochidia and encysted larvae on frozen and 5% formaldehyde fixed fish gills were performed under a light microscope (Carl Zeiss Axio Lab.A1, ZEISS, Jena, Germany) using ZEN software.

Prior to scanning electron microscopy SEM, suspended glochidia were immediately frozen at −80 °C and then freeze-dried. The images of the samples were obtained with a SEM Sigma VP ZEISS instrument (ZEISS, Jena, Germany) (10 kV, InLens detector) in the Core Facility Center “Arctic” of Northern Arctic Federal University, Arkhangelsk, Russia (unique identifier RFMEFI59417X0013). A platinum-palladium coating with a thickness up to 5 nm was applied to the surface by means of a Q150TES device (QUORUM) to enhance the image contrast.

### Mapping of the distribution ranges

The georeferenced distribution data set of Bolotov *et al*.^35^ was used for *M*. *dahurica*. The reliable records of two lenok species (*Brachymystax tumensis* and *B*. *lenok*) were collected from the body of available literature (Supplementary Table 3). The map has been created using ESRI ArcGIS 10 software (www.esri.com/arcgis).

### Ancestral host reconstruction analyses

We applied a Bayesian MCMC analysis implemented in RASP v. 3.2^89^. As an input tree data, we used the multi-locus fossil-calibrated phylogeny of Lopes-Lima *et al*.^7^, which was calculated on the basis of the most complete sequence data set (five markers: *COI* – 654 bp, *16S rRNA* – 475 bp, *18S rRNA* – 1778 bp, *28S rRNA* – 307 bp, and *H3* – 327 bp) sampled to date. Fish hosts of the Margaritiferidae were coded as follows: (a) non-salmonid hosts, and (b) salmonid hosts. The primary data on fish hosts has been collected from Lopes-Lima *et al*.^7^. The hosts of several species, i.e. *Gibbosula laosensis* (Lea, 1863), *G*. *crassa* (Wood, 1815), *Pseudunio homsensis* (Lea, 1865), and *P*. *marocanus* (Pallary, 1918), remain unknown. However, they most likely associated with non-salmonid fishes (Table 3), and we therefore assigned “b” code for these taxa. The analysis was computed with 500,000 generations (sampling every 100th generation) and 10 MCMC chains (temp = 0.1). Null distribution and two-host combination (i.e. “ab”) were not allowed. We applied a 10% burn-in to exclude the pre-convergence part of the simulation.

### Literature searching

The literature review was performed by searching through the ISI Web of Knowledge and Scopus databases using the following keywords: *Brachymystax lenok*, *Thymallus tugarinae*, Amur river fish, Primorski krai fish. Since these databases do not take into account the body of literature in Russian, we also looked for Cyrillic sources in RISC (Russian Index for Scientific Citation, https://elibrary.ru). Old publications were searched in libraries collections of the Russian State Library, Zoological Institute of the Russian Academy of Sciences, Russian Federal Research Institute of Fisheries and Oceanography, and the Berg State Research Institute on Lake and River Fisheries.

## Supplementary information

Fish hosts, glochidia features and life cycle of the endemic freshwater pearl mussel Margaritifera dahurica from the Amur Basin

## Data Availability

The sequences generated under this study are available from the GenBank database. Accession numbers for each specimen are presented in Table 1. The voucher specimens and fish host samples are available in the Russian Museum of Biodiversity Hotspots (RMBH), Federal Center for Integrated Arctic Research, Russian Academy of Sciences, Arkhangelsk, Russia.
