# Supplementary material for: Fish hosts, glochidia features and life cycle of the endemic freshwater pearl mussel Margaritifera dahurica from the Amur Basin
Source: Sci Rep. 2019 Jun 5;9:8300. doi: 10.1038/s41598-019-44752-9 (PMC6549177; doi:10.1038/s41598-019-44752-9)
Supplement: Supplementary file 1 — Fish hosts, glochidia features and life cycle of the endemic freshwater pearl mussel Margaritifera dahurica from the Amur Basin [file 41598_2019_44752_MOESM1_ESM.pdf]

SUPPLEMENTARY INFORMATION

# Fish hosts, glochidia features and life cycle of the endemic freshwater pearl mussel *Margaritifera dahurica* from the Amur Basin

Ilya V. Vikhrev*, Alexander A. Makhrov, Valentina S. Artamonova, Alexey V. Ermolenko, Mikhail Yu. Gofarov, Mikhail B. Kabakov, Alexander V. Kondakov, Dmitry G. Chukhchin, Artem A. Lyubas & Ivan N. Bolotov

*Corresponding author: vikhrevilja@gmail.com

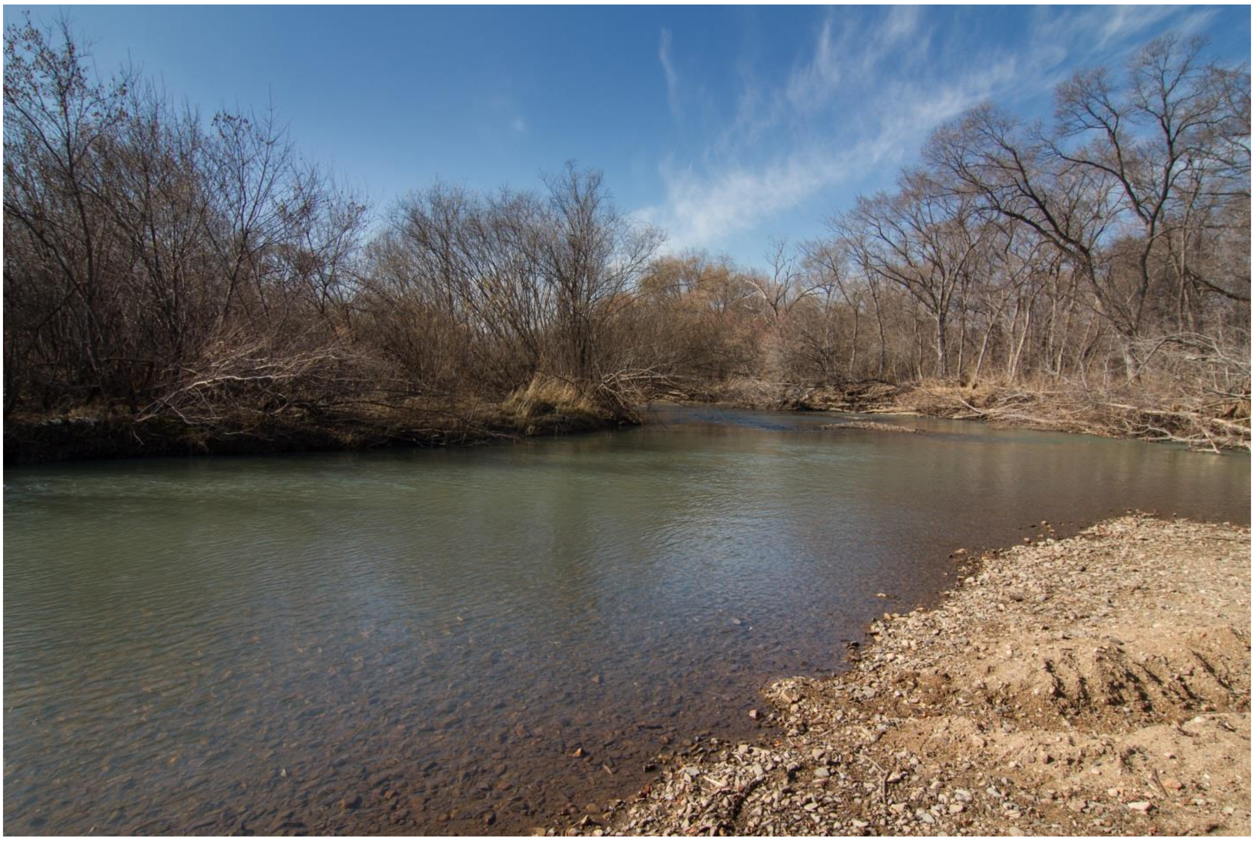

Headwater of the Komissarovka River (Khanka Lake Basin), a typical habitat of *Margaritifera dahurica* and its hosts: Lower Amur grayling (*Thymallus tugarinae*), sharp-snouted lenok (*Brachymystax lenok*), and blunt-snouted lenok (*B. tumensis*). (Photo: Ilya V. Vikhrev).

**Supplementary Table 1.** Comparison of glochidia size among freshwater pearl mussels in the beginning of metamorphosis ([1] with our adds)

| Species | Length (μm) | Height (μm) | Width (μm) | Reference |
|---|---|---|---|---|
| *Margaritifera dahurica* | 48.48-64.39 | 40.97-68.89 | 44.87-56.4 | Present study (*N* = 26) |
| *M. margaritifera* | 30.17-117.51 | n/a | n/a | [2] |
| *M. falcata* | 70-73 | 75-80 | n/a | [3] |
| *M. laevis* | 67.5-75 | n/a | n/a | [4] |
| *M. middendorffi* (= *M. togakushiensis*) | 65-75 | n/a | n/a | [4] |
| *Pseudunio auricularius* | 127-144 | 120-142 | 54-71 | [1] |
| *Cumberlandia monodonta* | 55 | n/a | n/a | [5] |

n/a – not available

**Supplementary Table 2.** Shell length of glochidia of *Margaritifera dahurica* during metamorphosis

| Sampling time | Shell length (μm) | |
|---|---|---|
| | **Mean ± SD** | **min-max** |
| End of August (right after releasing) (*N* = 26) | 57.42±3.49 | 48.48-64.39 |
| March (*N* = 114) | 93.84±2.87 | 41.09-188.04 |
| End of May (*N* = 5) | 150.66±10.2 | 89.36-189.1 |

**Supplementary Table 3.** Records of *Brachymystax* spp. and *Margaritifera dahurica* in the Amur Basin and adjacent river drainages of the Pacific Ocean (Russian part only)

| No. | Drainage | Lenoks | *M. dahurica* | References on the records of lenoks* |
|---|---|---|---|---|
| | **Major tributaries of the Amur river** | | | |
| 1 | Shilka | + | + | 6–10 |
| 2 | Zeya | + | + | 7,9,11,12 |
| 3 | Salasu | + | - | 11 |
| 4 | Amgun’ | + | - | 6,7,9,10 |
| 5 | Bureya | + | + | 7,9,13 |
| 6 | Ussuri | + | + | 6,7,9,10,14–20 |
| 7 | Machtovaya | + | - | 6 |
| 8 | Anui | + | - | 6,7,9,11,20 |
| 9 | Bol’shoi Never | + | - | 10 |
| 10 | Gorin | + | - | 10 |
| 11 | Tunguska | + | + | 9,21 |
| 12 | Bira | + | + | 21 |
| 13 | Gur | + | - | 9 |
| 14 | Argun’ | + | + | 10,12 |
| 15 | Arhara | + | + | 22 |
| 16 | Sungari | + | + | 23 |
| | **Separate river drainages of the Pacific Ocean** | | | |
| 17 | Uda | + | - | 7,24 |
| 18 | Tylakachan | + | - | 24 |
| 19 | Tygur | + | - | 24 |
| 20 | Ul’ban | + | - | 24 |
| 21 | Itkan | + | - | 24 |
| 22 | Usalgin | + | - | 24 |
| 23 | Iska | + | + | 25 |
| 24 | Psyu | + | - | 9 |
| 25 | My | + | - | 11 |
| 26 | Samarga | + | - | 7,20,26,27 |
| 27 | Edinka | + | - | 7,20,26,27 |
| 28 | Venykovka | + | - | 20,26,27 |
| 29 | Maksimovka | + | - | 20,26,27 |
| 30 | Kema | + | - | 26,27 |
| 31 | Taezhnaya | + | - | 26,27 |
| 32 | Serebryanka | + | - | 26,27 |
| 33 | Dzhigitovka | + | - | 26,27 |
| 34 | Rudnaya | + | - | 26 |
| 35 | Avvakumovka | + | - | 26 |
| 36 | Kievka | + | - | 26 |
| 37 | Partizanskaya | + | - | 28,29 |
| 38 | Suhodol | + | - | 28 |
| 39 | Shkotovka | + | - | 20,30 |
| 40 | Artemovka | + | - | 15 |
| 41 | Sedanka | + | - | 6,15 |
| 42 | Bogataya | + | - | 15 |
| 43 | Razdol’naya | + | + | 20,30 |
| 44 | Tumannaya | + | - | 29 |

*Records of *Margaritifera dahurica* were obtained from[31]. “+” – species present, and “-” – species absent. Numbers of rivers corresponds to those in the map (Fig. 1).

## Supplementary References


1. Araujo, R. & Ramos, M. A. Description of the glochidium of Margaritifera auricularia (Spengler 1793) (Bivalvia, Unionoidea). *Philos. Trans. R. Soc. B Biol. Sci.* **353,** 1553–1559 (1998).

2. Ieshko, E. P. *et al.* Freshwater pearl mussel Margaritifera margaritifera L. in Syuskyuyan Loki (Ladoga's lake basin). *Tr. Karel. Nauchnogo Cent. Ross. Akad. Nauk* **6,** 122–130 (2014).

3. Karna, D. W. & Millemann, R. E. Glochidiosis of Salmonid Fishes. III. Comparative Susceptibility to Natural Infection with Margaritifera margaritifera (L.) (Pelecypoda: Margaritanidae) and Associated Histopathology. *J. Parasitol.* **64,** 528 (1978).

4. Kobayashi, O. & Kondo, T. Comparative morphology of glochidia and juveniles between two species of freshwater pearl mussel Margaritifera (Bivalvia: Margaritiferidae) from Japan. *Venus* **65,** 355–363 (2007).

5. Howard, A. D. Some exceptional cases of breeding among the Unionidae. *Nautilus (Philadelphia).* **29,** 4–11 (1915).

6. Dorofeyeva, E. A. & Pripodina, V. P. *Catalog of specimens in the collection of the Zoological Institute, Russian Academy of Sciences. Osteichthyes, Salmoniformes. (Explorations of the fauna of the seas. v. 67).* (Zoological Institute of RAS, 2011).

7. Froufe, E., Alekseyev, S., Alexandrino, P. & Weiss, S. The evolutionary history of sharp- and blunt-snouted lenok (Brachymystax lenok (Pallas, 1773)) and its implications for the paleo-hydrological history of Siberia. *BMC Evol. Biol.* **8,** 40 (2008).

8. Gorlacheva, E. P. & Afonin, A. V. Features of distribution and biology of fishes in headwaters of the Onon river. *Environ. Coop. Russ. Mong. China* **2,** 53–58 (2011).

9. Antonov, A. L. Diversity of fishes and structure of ichthyocenoses in mountain catchment areas of the Amur Basin. *J. Ichthyol.* **52,** 149–159 (2012).

10. Nikolsky, G. V. *Fish of the Amur River basin.* (Publishing House of the USSR Academy of Sciences, 1956).

11. Alekseev, S. S. Morphoecological characteristics of the Lenock (Salmonidae, Brachymystax) from the Amur basin and from the river Uda. *Russ. J. Zool.* **62,** 1057–1067 (1983).

12. Taranets, A. Y. About fish and fishing in Noro-Selemzhinskom area (Zeya River basin). *Izv. TINRO* **12,** 71–77 (1937).

13. Antonov, A. L. Ichthyofauna of the upper part of Bureya River basin. *Vestn. Far East Branch Russ. Acad. Sci.* **3,** 49–59 (2007).

14. Semenchenko, A. Y. & Zolotukhin, S. F. Brief Review of Fish Fauna of the Bikin Nature Park (Ussuri River, Amur River Basin). *Biodivers. Environ. Far East Reserv.* **1,** 25–43 (2016).

15. Lindberg, G. U. & Taranets, A. Y. List of fishes of the Vladivostok State Museum. *Notes Vladivostok branch Russ. Geogr. Soc.* **4,** 221–266 (1929).

16. Besednov, L. N. & Kucherov, A. N. About the systematic position of the lenok of the genus Brachymystax of the river Iman. in *Zoological problems of Siberia. Materials IV of the meeting of zoologists of Siberia.* 220–221 (Nauka, 1972).

17. Bogutskaya, N. G. & Naseka, A. M. *Cyclostomata and fishes of Khanka lake drainage area*

*(Amur river basin). An annotated check-list with comments on taxonomy and zoogeography of the region.* (State Institute on lake and river fisheries; Zoological institute of the Russian Academy of Sciences, 1996).

18. Zolotuhin, S. P., Semenchenko, A. Y. & Belyaev, V. A. *Taimens and lenoks of the Russian Far East*. (Khabarovsk branch of TINRO, 2000).

19. Nazarenko, A. A. *Vertebrates of the Khankai Reserve and the lowland of the Khanka.* (Ltd. RIC «Idea», 2006).

20. Shedko, S. V. *Phylogenetic links of lenoks of genus Brachymystax (Salmonidae, Salmoniformes) and their specitations*. (Lambert Academic Publishing, 2012).

21. *Fauna of Bastak Nature Reserve.* (Publishing house of the Blagoveshchensk State pedagogical institute, 2012).

22. *Fish in the reserves of Russia*. (The Partnership of Scientific Publications of KMK, 2010).

23. Li, S. Studies on the distribution of the Salmonid fishes in China. *Chinese J. Zool.* **1,** 34–37 (1984).

24. Zolotukhin, S. F., Makhinov, A. N. & Kanzeparova, A. N. Features of morphology and hydrology for spawning rivers at the northwestern coast of the Okhotsk Sea. *Notes of TINRO* **176,** 139–154 (2014).

25. Taranets, A. Y. About zoogeography of the Amur transitional area: a case study of freshwater ichtyophauna. *Lett. Far East. Branch AS USSR* **32,** 91–115 (1938).

26. Shedko, S. V. List of Cyclostomes and Freshwater Fish in the Coast of Primorye. *Readings Mem. Vladimir Yakovlevich Levanidov* **1,** 229–249 (2001).

27. Kolpakov, E. V. & Kolpakov, N. V. Ichthyofauna of inland waters in Northern Primorye. *J. Ichthyol.* **43,** 708–712 (2003).

28. Dulkeit, G. D. About the fauna of freshwater fish of the southern Sikhote-Alin (Ussuri region). *Yearb. Zool. Museum USSR Acad. Sci.* **28,** 9–24 (1927).

29. Taranets, A. Y. Freshwater fishes of the north-western part of the Japan Sea basin. *Notes Zool. Inst. AS USSR* **4,** 485–540 (1936).

30. Shedko, S. V. & Shedko, M. B. A new data on freshwater ichthyofauna of the south of the Russian Far East. in *Vladimir Ya. Levanidov's Biennial Memorial Meetings. V. 2.* 319–336 (Dalnauka, 2003).

31. Bolotov, I. N. *et al.* Taxonomy and distribution of freshwater pearl mussels (unionoida: Margaritiferidae) of the Russian far east. *PLoS One* **10,** (2015).
